# Epidemiological characteristics, clinical manifestations and laboratory findings in 850 patients with brucellosis in Heilongjiang Province, China

**DOI:** 10.1186/s12879-019-4081-5

**Published:** 2019-05-20

**Authors:** Wenhui Jiang, Jiwang Chen, Qian Li, Lisheng Jiang, Yanxin Huang, Yinghua Lan, Yongguo Li

**Affiliations:** 1 Department of Infection Diseases, the First Affiliated Hospital of Harbin Medical University, Harbin, 150001 Heilongjiang China; 2 The Second Hospital of Daqing, Daqing, 163000 Heilongjiang China

**Keywords:** Brucellosis, Epidemiology, Clinical characteristics, Complications, Laboratory findings, Relapse

## Abstract

**Background:**

Brucellosis has extensive clinical spectrum, clinicians have insufficient understanding of the disease, and the misdiagnosis rate is still high. By collecting and analyzing the clinical characteristics of patients with brucellosis in Heilongjiang Province to provide guidance and reference for clinicians to make timely diagnosis and treatment.

**Methods:**

The demographic and epidemiological characteristics, clinical features, complications, laboratory findings were retrospectively evaluated in 850 brucellosis patients admitted in the Department of Infectious Diseases of the First Affiliated Hospital of Harbin Medical University and the Second Hospital of Daqing from 2012 to 2017.

**Results:**

Of the 850 patients, the most common clinical manifestations were fever (93.3%), joint pain (69.8%), sweating (45.2%), fatigue (38.6%), and splenomegaly (34.0%). Peripheral arthritis, spondylitis and epididymal-orchitis were the common complications. Of the 398 patients who were followed up and completed treatment, 22 (5.5%) had relapse.

**Conclusions:**

Brucellosis is a multisystem disease with diverse clinical manifestations. In areas where brucellosis is endemic, the possibility of the disease should be considered in patients with unexplained fever and joints pain. In addition, the high rate of relapse is mainly due to the misdiagnosis of complications, so local CT or MRI examination is necessary for patients with joint pain and low back pain. Timely diagnosis, early detection of complications are essential to improve the prognosis and reduce relapse.

## Background

Brucellosis, a zoonosis caused by bacteria of the *Brucella spp*, is still an important public health problem in the world, especially in many developing countries. The disease is transmitted to humans through close contact with domestic animals in addition to the cibation of raw dairy products and infected meat from livestock [1]. In China, the incidence of brucellosis infections has increased in recent years. Nationwide surveillance data showed that the overall incidence rate of human brucellosis increased from 0.92 /100,000 in 2004 to 4.2 /100,000 in 2014 [2–4]. Brucellosis is mainly prevalent in the Northeast, Northwest, Inner Mongolia, qinghai-tibet plateau and other pastoral areas [5]. In recent years, the range of high incidence areas had also tended to move southward. At present, the prevalence of brucellosis is still increasing in the old epidemic areas, and new epidemic areas are emerging gradually [3, 6]. This epidemic trend is mainly caused by the increase of livestock breeding through backyard farming practices, insufficient supervision and epidemic prevention, the development of tourism, changes in dietary habits and weak awareness of health and self-protection. Located in the northeastern part of China, Heilongjiang Province is one of the provinces with many agricultural and animal husbandry activities, the incidence of brucellosis is also on the rise and is fast becoming a major public health problem.

Brucellosis has a broad clinical spectrum, and its clinical features depend on the stage of the disease and the organs/ systems involved [7]. High fever, hydrolysis, fatigue and arthralgia of the large joints are the main symptoms of brucellosis. Brucellosis can also mimic various multisystem disease and cause multiple complications, such as those of the musculoskeletal system, hematological system, digestive system, nervous system and urinogeenital system. Common complications of brucellosis include arthritis, spondylitis, meningitis, epididymal-orchitis, pneumonia, etc. The most frequent of these is the musculoskeletal system involved. This wide clinical overlap with other diseases frequently leads to misdiagnosis and treatment delays [8]. Furthermore, the lack of epidemiological history, slow growth rate in blood cultures, asymptomatic infections, and chronic infections with atypical symptoms are common causes of delayed diagnosis and misdiagnosis. Brucellosis is a natural focus disease, but as the epidemic area of brucellosis gradually expands, the lack of understanding of brucellosis by clinicians in some non-endemic areas is also an important reason for misdiagnosis. Relapse is also one of the characteristic features of human brucellosis. The relapsed patients not only bear the pain again, but also increase the financial burden. Mastery of the clinical features of the disease, timely diagnosis and treatment will greatly improve the patient’s prognosis.

In this study, the clinical data of 850 patients were analyzed retrospectively to provide guidance and reference for clinician.

## Methods

The clinical data of 850 brucellosis patients who with or without complications were admitted to the department of infectious diseases of the First Affiliated Hospital of Harbin Medical University and the Second Hospital of Daqing from January 2012 to October 2017 were retrospectively analyzed. Clinical data included demographic and epidemiological characteristics, clinical manifestations and complications, physical examination findings, laboratory and imageological findings, and relapse. Most of patients’ data was obtained from electronic medical records, and patients were followed up by telephone to record relapsed cases.

The diagnosis of brucellosis was based on epidemiological data, clinical features, positive serum agglutination tests(tiger red plate agglutination test were positive or suspicious. The titer of the standard tube agglutination test was ≥1:100 or the duration of the course more than 1 year was ≥1:50) and/or blood, bone marrow, cerebrospinal fluid (CSF) and other body fluids cultures. According to the duration of clinical symptoms, patients were divided into two groups: acute phase (less than 6 months) and chronic phase (more than 6 months) [9].

A detailed history and physical examination was undertaken for each brucellosis case at the beginning of hospitalization. The standard tube agglutination test, blood cell analysis, routine biochemistry parameters, myocardial enzyme detection, and routine urinalysis were measured at admission. Double sets of blood cultures were collected for the patients before the use of antibiotics. Ultrasonography examination of abdomen and urinary systems was also performed at admission. Moreover, for some cases with suspected complications, CT and/or MRI of suspicious parts, echocardiography, scrotal doppler ultrasonography, and other imaging examinations were carried out. Some invasive inspections such as lumbar puncture and bone marrow aspiration were also performed when necessary.

Fever of unknown origin was defined as body temperature ≥ 38.3 °C on three or more occasions and a duration of illness of at least 3 weeks, with no diagnosis made despite 1 week of inpatient examination [10]. Osteoarticular involvement was considered if there were any signs of inflammation in any joint combined with imaging abnormalitie. Spondylitis was diagnosed using magnetic resonance imaging (MRI) or computerized tomography (CT). Epididymo-orchitis was diagnosed by discovering sore pain of scrotum, testicle and epididymis, with positive ultrasound findings. The diagnosis of meningitis included positive results of Brucella in cerebrospinal fluid culture and/or neurological symptoms (headache, dizziness, stiff neck) and abnormal cerebrospinal fluid examination. Nerve involved included nerve conduction abnormalities, sensory disturbance, and changes in mental or mood status also involved. Relapse was defined as the reappearance of symptoms or a positive blood culture within 6 months after therapy [1].

## Results

### Demographic and epidemiological characteristics

Of the 850 patients, 633 (74.5%) were male, 437 (51.4%) patients were farmers and herdsmen. In terms of epidemiological history, 599 (70.5%) exposured to cattle or sheep, mostly sheep. 781 (91.9%) cases were in acute phase and 69 (8.1%) cases were in chronic phase (Table 1).

**Table 1:** Demographic and epidemiological characteristics of patients with brucellosis (total, No. = 850)

Variable	No.(%)
Sex	
Male	633 (74.5)
Female	217 (25.5)
Residence	
Urban	177 (20.8)
Rural	673 (79.2)
Age group	3–87
Mean age	46.0 ± 13.4
0-17 years	19 (2.2)
18–40 years	239 (28.1)
41–65 years	544 (64.0)
>65 years	48 (5.7)
Mean hospitalization time (day)	12.1 ± 2.8
The mean time from symptom onset to hospitalization	59.2 ± 99.2
Admission date (month)	
Spring (3–5)	273 (32.1)
Summer(6–8)	303 (35.7)
Autumn(9–11)	137 (16.1)
Winter (12–2)	137 (16.1)
Medical history	
Exposure and farming of cattle or sheep	599 (70.5)
Consumption history of raw meat or dairy	76 (8.9)
Unknown	175 (20.6)
Occupation	
Farmer and herdsman	437 (51.4)
Worker	118 (13.9)
Vagrant	88 (10.4)
Student	16 (1.9)
Veterinarian	3 (0.3)
Other	188 (22.1)
Staging	
Acute	781 (91.9)
Chronic	69 (8.1)

### Clinical characteristics and complications

The most frequent symptoms were fever (93.3%), arthralgia (69.8%), hyperhidrosis (45.2%), fatigue (38.6%), and anorexia (29.5%). In patients with fever, 395 were diagnosed with fever of unknown origin. For complications, arthrophlogosis (14.2%) and spondylitis (13.1%) were found in all cases, and other common complications included epididymoorchitis (5.9%), pneumonia (4.7%), meningitis (1.1%) (Table 2).

**Table 2:** Clinical characteristics and complications of patients with brucellosis

Variable	No.(%)
Symptoms	
Fever	793 (93.3)
Hyperhidrosis	384 (45.2)
Fatigue	328 (38.6)
Arthralgia	593 (69.8)
Headache and Dizziness	153 (18.0)
Anorexia	251 (29.5)
Lose weight	68 (8.0)
Physical examinations	
Hepatomegaly	73 (8.6)
Splenomegaly	289 (34.0)
Hepatosplenomegaly	55 (6.5)
Lymphadenectasis	91 (10.7)
Complications	
Arthrophlogosis	121 (14.2)
Spondylitis	111 (13.1)
Lumbar involved	105
Thoracic vertebra involved	6
Cervical vertebra involved	9
Sacral vertebrae involved	4
Epididymo-orchitis	50 (5.9)
Pneumonia	40 (4.7)
Meningitis	9 (1.1)
Focal abscess	5 (0.6)
Soft tissue inflammation	8 (0.9)
Nerve involved	3 (0.4)
Infective endocarditis	1 (0.1)

### Laboratory findings

Table 3 shows the results of laboratory examinations. Of 430 patients who underwent blood culture examination, of which positive blood culture were 208 (48.4%). Common hematological changes included leukopenia (17.9%), lymphocythaemia (34.7%), hypoeosinophilia (26.9%), anemia (25.5%). 494 (58.1%) had elevated levels of aminotransferase.

**Table 3:** Laboratory fingdings of patients with brucellosis

Variable	No.(%)
Positive culture	208 (48.4)
Negative culture	222 (51.6)
WBC count(×10^9/L)	
Hypoleucocytosis(<4)	152 (17.9)
Leukocytosis (>10)	48 (5.6)
Lymphocytosis (>40%)	295 (34.7)
Hypoeosinophilia (<0.05%)	229 (26.9)
Anemia (hemoglobin: male<130 g/L, female<110 g/L)	217 (25.5)
Mild	205
Medium	12
Severe	0
Platelet count (×10^9/L)	
Thrombocytopenia(<98)	78 (9.2)
Thrombocytosis (>300)	103 (12.1)
Pancytopenia	23 (2.7)
Elevated aminotransferases(>40 U/L)	494 (58.1)
Creatinine increased(44-97umol/L)	23 (2.7)
CK increased(26–140 U/L)	52 (6.1)
α-HBDH increased (72–182 U/L)	264 (31.1)

Note: *WBC count* white blood cell count, *CK* creatine kinase, *α-HBDH* alpha- hydroxybutyric dehydrogenase

### Relapse

Among the 850 patients, 518 patients were followed up successfully. However, only 398 cases completed the treatment as prescribed by the doctor. The main reasons for the 120 patients who did not complete the prescribed treatment were change of hospital (often to other specialist hospital and clinics) and change of drugs to Chinese medicine or other medicines with unknown ingredients that have not been verified to have therapeutic effect, these cases were excluded. In 398 patients, 22 (5.5%) had relapses within 6 mouths after therapy. Of the 22 patients who relapsed, 15 patients raised sheep or had a clear exposure history of sheep, and 2 patients were cattle farmers. Nine patients were in a chronic stage. Nineteen patients had symptom of joint pain, 5 of which were examined by joint magnetic resonance imaging (MRI), 3 patients underwent joint X-ray only, and 11 patients with joint pain didn’t undergo imaging examination.

## Discussion

Human brucellosis remains one of major public health issues in China. The epidemiology of human brucellosis has tremendously changed in our country owing to the changing hygienic and socioeconomic conditions, and the significant increase in international and domestic tourists [11]. Presently, human brucellosis is epidemic in 25 out of the 32 provinces or autonomous regions of China [12]. With the epidemic area of brucellosis gradually expanded, delayed diagnosis and misdiagnosis are more common, especially in non-endemic areas. Wang et al. [11] reported that out of 141,604 brucellosis cases, 26.98% were diagnosed within 7 days of symptom onset, 43.83% within 14 days, and 2.39% longer than 6 months. Two thousand sixty cases were collected from brucellosis clinics, 57.62% of the patients had been misdiagnosed or suspected of having other diseases with similar symptoms. Zheng et al. [13] reported the latest statistics of the misdiagnosis rate, in which 2287 cases were misdiagnosed at the first visit, and the misdiagnosis rate was 62.5%, and most of these cases were from non-pastoral areas. Misdiagnosis and delayed diagnosis can increase the risk of chronic disease. Chronic brucellosis can cause systemic nonspecific symptoms, such as neurosis, and chronic fatigue syndrome which can cause physical damage of the skeletal muscle system mostly in large joints and tendon contracture.

In this study, 70.5% of patients were infected by direct contact with livestock, the vast majority was sheep, so it is speculated that B. melitensis is probably the most common strain in this area, followed by *B. abortus*, but this speculation lacks exact etiological evidence to confirm at present. However, previous studies on the pathogenic analysis of brucellosis in humans and animals revealed the main species of brucella in China, it was found that sheep species (B. melitensis) was the most common, followed by cattle species (B. abortus) and pig species (B. suis) [12, 14]. The majority of infected people were middle-aged men between the ages of 41 to 65. The major reason is that this group is the main labor force of family and society and has more chances of access the livestock. Most of the patients (67.8%) came to the hospital for treatment in spring and summer, because cows and sheep mostly give birth in the spring, hence the close contact with ewes and lambs may be the main cause of high number of patients in these seasons. The proportion of patients with chronic brucellosis was 8.1%, this ratio is similar to previous studies [15–17].

Brucellosis is a disease involving multiple systems with diverse clinical manifestations. The disease is characterized by are fever, hydrolysis, anorexia, arthralgia, fatigue and hepatosplenomegaly [18]. However, brucellosis can also show some atypical symptoms which prompt patients to seek treatment, such as symptoms of the digestive system (abdominal pain, nausea and vomiting, anorexia, jaundice, etc.) [19], nervous system (dizziness and headache, neck stiffness, cranial nerve damages, etc.) [20], respiratory system (dry cough, dyspnea, chest pain, etc.) [21], cardiovascular system (chest pain, pericardial friction rub, heart murmur, etc.). In this study, fever, arthralgia, hyperhidrosis and fatigue were common symptoms. The proportion of patients with fever was 93.3%, this percentage is higher compared to previous studies [1, 15–17, 22, 23]. The reason is that cases enrolled in our study were collected from the department of infectious disease, the majority of patients were admitted to hospital with fever of unknown cause. Some patients with atypical symptoms were transferred to other departments, this was a limitation of this study because some atypical symptoms were not studied and described. Among them, 395 cases were admitted to the department of infectious diseases for fever of unknown origin. Due to the diversity of symptoms of brucellosis and the lack of attention of doctors in the grassroots medical units, which is often misdiagnosed as common bacterial infection. However, brucellosis has the characteristics of undulating fever, they were mistakenly believed to be getting better in the intermittent period of fever. After repeated fever, they were admitted to the hospital with unexplained fever. In addition, owing to China’s national conditions, patients require hospitalization in order to relieve symptoms quickly, find out the causes of recurrent fever and start medicare to reduce costs, so patients with or without complications can be hospitalized in China. 45.2% of patients had the symptoms of sweating, this percentage is lower compared to previous studies in China and abroad [1, 15–17, 22, 23]. It is likely that the clinician ignored the symptoms of sweat during history-taking. In addition, due to the abuse of antipyretic drugs, some patients were unaware of the drug-induced sweating and the hyperhidrosis of brucellosis itself. The frequencies of hepatomegaly, splenomegaly and lymphadenopathy were 8.6, 34.0, and 10.7%, respectively. This clinical feature is an indication of its predilection for reticuloendot- helial system (RES) [24]. The occurrence of complications is also related to this characteristic of brucella. Brucella spreads to tissues rich in elements of reticuloendothelial system, such as the joints, genitourinary system, central nervous system, cardiovascular system and respiratory system. Osteoarticular complications are the most common complications of brucellosis. A previous systematic study showed that arthralgia was common in brucellosis, affecting 65% patients overall, whereas arthritis affected only 26% patients, and spondylitis were detected in 12–36% adults [25]. In this study, the probability of joint pain was 69.8%, and the incidence of arthrophlogosis and spondylitis was 14.2 and 13.1%, respectively. This result is consistent with the whole situation of brucellosis. Peripheral joint pain mainly occured in large joints such as the hip and knee, few patients had pain in their fingers and toes. In patients with spondylitis, the most vulnerable part was the lumbar spine, and it may lead to the formation of a local abscess near the spine, causing severe pain during exercise. The abscess must be treated surgically in necessity to eliminate the symptoms of oppression when medical treatment is ineffective. Other rare clinical complications of brucellosis include lung abscess, chesthilar lymphadenopathy, acute pericarditis, myocarditis, endarteritis, thrombophlebitis, mycotic aneurysms, erythematous papular lesions, and chorea [7]. But none of these complications were observed in this study.

Brucellosis is a multisystem disease that is also manifested in abnormalities in laboratory examinations of multiple systems. The most common findings involving the digestive system are abnormal liver function, which is mainly characterized by the increase of transaminase. Brucellosis involves the liver in varying ways, ranging from benign subclinical increases in serum aminotransferase levels to ominous chronic suppurative disease [26]. Brucellosis can also cause some changes in the hematological system. Mild anemia, leukopenia, thrombocytopenia, and pancytopenia which are relatively common. Hypersplenism, hemophagocytic syndrome, hypoplasia, and granulomatous inflammation of the bone marrow, and destruction of immune system are likely causes of these abnormalities in the peripheral blood [24, 27]. In this study, the frequencies of anemia, leukopenia, thrombocytopenia, and pancytopenia were 25.5, 17.9, 9.2, and 2.7%, respectively. It’s worth mentioning that 26.9% of the patients in this study had decreased eosinophils. Eosinophils are decreased in typhoid fever, paratyphoid fever, severe tissue injury after surgery, and during the use of adrenal cortex hormone or adrenocorticotrophic hormone. Brucellosis antigen shares some common components with the antigen of typhoid fever and paratyphoid fever [28, 29]. Therefore, the decrease or even disappearance of eosinophils may have some implications for early diagnosis of brucellosis.

Relapses are common (5–15% cases) and frequently result from missed diagnosis of complications, poor compliance with a prolonged course of therapy, inappropriate antibiotic drugs, or improper treated focal infection [19]. In this study, 22 (5.5%) patients had relapse within 6 months after treatment. Of these, 17 were considered to be uncomplicated at the time of treatment. In 22 patients with relapse, 19 patients had symptom of joint pain before they first start treatment, but the majority of those did not receive imaging examination. This phenomenon is related to current medical situation in China, where medical services are difficult and expensive. So patients refuse imaging examination, which leads to joint damage undiscovered. In addition, clinicians don’t pay enough attention to the occurrence of some clinical symptoms, the detection rate of complications was low by ignoring some necessary imaging examination, so that the course of treatment for these patients remains at the standard 6–8 weeks, treatment is insufficient, leading to relapse. Magnetic resonance imaging (MRI) is the most useful method for diagnosis, assessment of arthral or spinal brucellosis. In conclusion, patients with joint pain and long duration should be examined by joint or spine MRI with the aim of detecting joint complications as early as possible. During the follow-up period, we learned that two patients had a relapse for many times, their had clearly become chronic and difficult to treat. Chronic brucellosis can cause severe pain, difficulty in walking or even paralysis which seriously reduces the quality of life of patients. We believe that there is more chronic brucellosis than it is presented in this study. Hence, timely diagnosis and treatment, early recognition of complicated cases is imperative to reduce chronic disease and relapse.

## Conclusions

Human brucellosis is a multisystemic disease with a broad clinical spectrum which is one of the main reasons of high rate of delayed diagnosis and misdiagnosis. The foundation of clinical diagnosis depends on taking a detailed history and paying careful attention to epidemiological data. The high relapse rate is mainly due to the misdiagnosis of complications, so local CT or MRI examination is necessary for patients with joint pain and low back pain.

Clinicians should fully understand the clinical characteristics of brucellosis, and timely and effective diagnosis and treatment are essential to improve the prognosis of patients with brucellosis and reduce relapse. In areas where brucellosis is endemic, the possibility of the disease should be considered in patients with unexplained fever and joint pain.

## Abbreviations

B. melitensis: Brucella melitensis
B.abortus: Brucella abortus
B.suis: Brucella suis
CK: Creatine kinase
CSF: Cerebrospinal fluid
CT: Computed tomography
MRI: Magnetic resonance imaging
RES: Reticuloendothelial system
WBC: White blood cell
WHO: the World Health Organization
α-HBDH: alpha-hydroxybutyric dehydrogenase
